# Comparison of postprocessing metrics in multimetabolic APT-weighted CEST and 2-deoxy-D-glucose-CEST-MRI for differentiating breast cancer subtypes in a murine model

**DOI:** 10.1186/s41747-025-00665-z

**Published:** 2026-01-19

**Authors:** Daniela Prinz, Silvester J. Bartsch, Joachim Friske, Martin Krššák, Daniela Laimer-Gruber, Thomas H. Helbich, Katja Pinker

**Affiliations:** 1 Division of Molecular and Structural Preclinical Imaging (PIL), Department of Biomedical Imaging and Image-guided Therapy, Medical University of Vienna, Vienna, Austria; 2 Division of Endocrinology and Metabolism, Department of Medicine III, Medical University of Vienna, Vienna, Austria; 3 Division of Breast Imaging, Department of Radiology, Columbia University Vagelos College of Physicians and Surgeons, New York, NY USA

**Keywords:** Breast neoplasms, Disease models (animal), Magnetic resonance imaging, Mice, Molecular imaging

## Abstract

**Background:**

Chemical exchange saturation transfer (CEST)-magnetic resonance imaging (MRI), particularly amide proton transfer-weighted (APTw)-CEST and 2-deoxy-D-glucose-CEST, holds promise for noninvasive molecular breast cancer (BC) characterization. However, quantification remains challenging due to field inhomogeneities, overlapping exchange pools, and the limited robustness of conventional metrics such as the magnetization transfer ratio asymmetry (MTR_asym_). This study evaluates four CEST postprocessing metrics—MTR_asym_, Lorentzian amplitudes, MTR relaxation exchange (MTR_REX_), and apparent exchange-dependent relaxation (AREX)—for their diagnostic performance in differentiating BC subtypes using endogenous APTw-CEST and exogenous 2-deoxy-D-glucose-CEST in a murine BC xenograft model of Luminal A, human epidermal growth factor receptor 2 (HER2)+, and triple-negative tumors.

**Materials and methods:**

Metabolic CEST-MRI was performed *in vitro* on protein and 2-deoxy-D-glucose phantoms and *in vivo* in a murine BC model. Imaging was conducted at 9.4 T with 120 frequency offsets from +6 to -6 ppm. MTR_REX_ and AREX were derived via Lorentzian fitting using tailored five-pool models. Statistical comparisons across subtypes were performed per metric.

**Results:**

In APTw-CEST, MTR_REX_ and AREX significantly distinguished Luminal A from HER2+ (*p* ≤ 0.027) and Luminal A from triple-negative (*p* ≤ 0.006) tumors. Lorentzian amplitudes differentiated Luminal A from triple-negative (*p* = 0.019), while MTR_asym_ showed no separation. In 2-deoxy-D-glucose-CEST, only AREX distinguished Luminal A from HER2+ tumors (*p* = 0.017).

**Conclusion:**

Advanced metrics, particularly MTR_REX_ and AREX, improve metabolic CEST-MRI for BC subtyping in a murine preclinical model, while MTR_asym_ is inadequate for this purpose.

**Relevance statement:**

Our findings underscore the importance of applying advanced postprocessing metrics to metabolic CEST-MRI for improved noninvasive BC characterization in a murine preclinical model.

**Key Points:**

- Advanced multimetabolic APTw-CEST and 2-deoxy-D-glucose-CEST postprocessing metrics allowed adequate preclinical murine BC subtyping.
- AREX showed potential for 2-deoxy-D-glucose-CEST in tumor characterization; however, APTw-CEST remains superior.
- MTR_asym_ failed to distinguish between tumor subtypes in CEST-MRI.

**Graphical Abstract:**

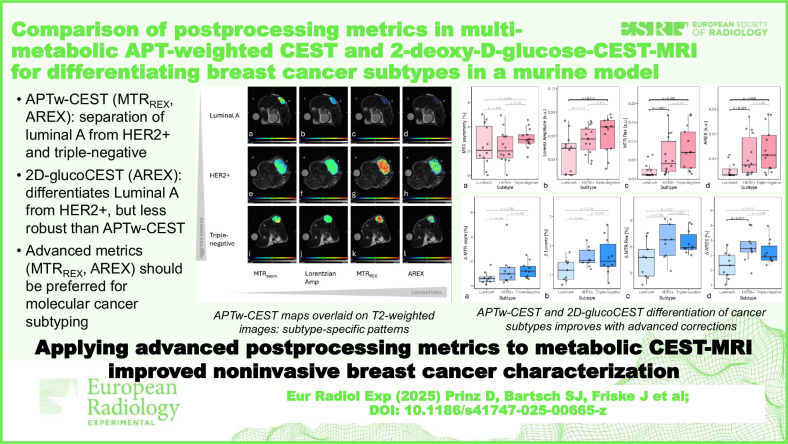

## Background

Chemical exchange saturation transfer (CEST)-magnetic resonance imaging (MRI) is a noninvasive metabolic imaging technique that relies on the exchange of selectively saturated solute protons with bulk water protons, leading to a detectable reduction in the water signal [1–3]. CEST-MRI enables imaging of both endogenous and exogenous solutes [4, 5].

Endogenous CEST includes several types, among them amide proton transfer-weighted (APTw)-CEST (-NH_2_) for imaging intracellular proteins and pH, glycoCEST (-OH, -NH) for glycogen, gagCEST for glycosaminoglycans in cartilage (-OH and -NH), and gluCEST (-NH_2_) for glutamate in the brain. Exogenous CEST applications include iopamidol-based acidoCEST for pH imaging (-OH) and glucoCEST to image glucose metabolism (-OH) [6]. For cancer imaging [7–10], the endogenous APTw-CEST [11–16] and glucoCEST [17–23] are promising as they provide insights into cancer biology. APTw-CEST allows imaging of highly proliferating tissues through the elevated signal originating from mobile proteins. Notably, glucoCEST, particularly 2D-glucoCEST using 2-deoxy-D-glucose (2-DG), is of interest as well, as it allows assessment of tumor glucose uptake similar to [^18^F]-fluorodeoxyglucose ([^18^F]FDG)-positron emission tomography (PET). Moreover, 2D-glucoCEST offers a radiation-free alternative with higher spatial resolution.

CEST-MRI implementation and quantification remain challenging due to artifacts arising from field inhomogeneities, sensitivity to motion, spillover from other exchange pools and complex postprocessing [24]. Most often, the CEST spectrum is quantified by applying a simple magnetization transfer ratio asymmetry (MTR_asym_) analysis, which is known to be affected by the intrinsic asymmetry of the spectrum influenced by the nuclear Overhauser effect (NOE). Advanced metrics that rely on Lorentzian fitting have been proposed to improve quantification accuracy [13, 25–31]. While extensively applied in APTw-CEST, their potential in glucoCEST remains largely unexplored. Although clinical APTw-CEST studies in breast cancer (BC) have reported variable absolute values, most indicate higher signals in more aggressive subtypes. Here, we employ advanced quantification metrics to improve specificity beyond conventional MTRasym [32–38].

This study aims to evaluate the performance of advanced CEST postprocessing metrics—originally developed in APTw-CEST—when applied and adapted to accommodate 2D-glucoCEST with a particular focus on distinguishing between three BC molecular subtypes in a well-established xenograft model. To achieve this, four CEST quantification metrics—MTR_asym_, Lorentzian amplitudes, MTR relaxation exchange (MTR_REX_), and apparent exchange-dependent relaxation (AREX)—are applied and compared for both APTw-CEST and 2D-glucoCEST across adjusted phantoms and experimental murine BC xenograft models.

## Materials and methods

### *In vitro* experiments

Phantom measurements were performed as a quality control step to assess z-spectra, fit and contrast-to-noise ratio (CNR). APTw-CEST z-spectra were evaluated on egg white + phosphate-buffered saline (PBS) phantoms at five egg white concentrations (20%, 40%, 60%, 80%, 100%) submerged into a vial containing 3% agarose + PBS to improve shimming and reduce vibrations. Egg whites are commonly used for *in vitro* APTw-CEST measurements [39]. For 2D-glucoCEST assessment, phantoms of 2-DG + PBS at five 2-DG concentrations were produced (4.5 mM, 9 mM, 13.5 mM, 18 mM, 22.5 mM) and submerged in a vial containing 3% agarose + PBS.

### *In vivo* experiments

Animal experiments were conducted in accordance with the Austrian Federal Ministry of Education, Science and Research and the Intramural Committee for Animal Experimentation of the Medical University of Vienna. Experiments complied with the Directive 2010/63/EU and adhered to Animal Research: Reporting of *in vivo* Experiments (ARRIVE) guidelines [40]. Additional details on animal housing, monitoring procedures, and humane endpoint criteria are provided in the Supplementary Material.

We utilized a well-established BC xenograft model of three different human BC subtypes for all 2D-glucoCEST and APTw-CEST measurements. The three BC cell lines with varying levels of aggressiveness were obtained from the American Type Culture Collection. This included the less aggressive Luminal A (MCF-7) cells, which are estrogen and progesterone hormone receptor positive, the more aggressive human epidermal growth factor receptor 2 (HER2) + SKBR-3 cells (which exhibit amplified and overexpressed *HER2/neu* while lacking hormone receptors, and contain a TP53 mutation), and the most aggressive triple-negative MDA-MB-231 cells (which lack both hormone receptors and HER2). Cells were grown under standard cell culture conditions (humidified air, 37 °C, 5% CO_2_) according to a protocol previously proposed by Bartsch et al [41]. Luminal A cells were kept in Roswell Park Memorial Institute‒RPMI medium, while HER2+ and triple-negative cells were maintained in Dulbecco’s Modified Eagle Medium‒DMEM, both supplemented with 10% fetal bovine serum.

Female athymic BALB/c-derived nude mice were injected subcutaneously into the right flank with 1 $\times$ 10^7^ Luminal A cells (*n* = 16), 3 $\times$ 10^6^ HER2+ cells (*n* = 17) and 3 $\times$ 10^6^ triple-negative cells (*n* = 25) under constant anesthesia using isoflurane (Zoetis Österreich GmbH). Tumors were allowed to grow for approximately 2 weeks. One week prior to MCF-7 cell inoculation, isoflurane-anesthetized mice were implanted subcutaneously in the neck region with low-dose estrogen pellets (0.36 mg/day, 60-day release, Innovative Research of America) using a 10-G precision trocar [42]. Tumor growth was monitored by regular visual inspection and palpation, with MRI performed either 2‒3 weeks post-inoculation or when tumors approached a maximum diameter of 1 cm. For *in vivo* MRI measurements, mice were placed into an induction box with 3% isoflurane mixed with medical air to initiate anesthesia. They were then positioned on a heating pad (37 °C) under continued anesthesia through a nose cone. A 30-G needle containing heparinized saline solution (0.9%) with a 10 cm-long catheter was injected into the lateral tail vein. The mice were then transferred into the MRI cradle with continuous anesthesia (0.5‒1.5%) to keep the respiratory rate at 60–80 breaths per minute. Breathing rate was monitored, and body temperature was maintained. The tail vein catheter was connected to a 1-mL syringe mounted to an infusion pump (PHD 2000, Harvard Apparatus) to enable remote-controlled injection. A 2-m tubing line (Tycon® Flexible Plastic Tubing) with an inner diameter of 0.25 mm was prefilled with a 2-DG solution (Sigma Aldrich, Merck KGaA). 150 $\mu$L of 2-DG, corresponding to a dose of 0.5 g/kg body weight, was injected, adjusted to the weight of the animal. Infusion was initiated in three steps to enhance tolerability: 0.04 mL at 0.03 mL/min, followed by 0.06 mL at 0.015 mL/min and 0.05 mL at 0.03 mL/min. Total anesthesia time was limited to 120 min. After *in vivo* PET/MRI, animals were euthanized using 300 mg/kg pentobarbital intravenously injected (Release, WDT) and tumors collected.

### MRI acquisition

All MRI measurements followed the protocol outlined previously by Bartsch et al [41] and were performed using the same setup for both *in vitro* and *in vivo* measurements. MRI scans were performed on a 9.4 T preclinical Biospin 30/94 USR scanner (Bruker) equipped with a BGA 20 gradient system and PET-optimized 35 mm ^1^H volume coil. The system operated with ParaVision v360.33 software (Bruker BioSpin GmbH).

After scout images, T2-weighted anatomic images were acquired using a rapid acquisition with relaxation enhancement (RARE) sequence (repetition time (TR) 2,000 ms, echo time (TE) 35 ms, spatial resolution 0.102 × 0.107 mm^2^, slice thickness 1 mm, matrix size 320 × 320, number of slices 10, acquisition time (TA) 1:20 min:s). Based on those anatomic images, for *in vitro* images, a slice that did not contain air, and for *in vivo* images, the axial slice with the largest tumor cross section was chosen for all following single slice scans (WASSR, CEST, T1 map). Water saturation shift referencing (WASSR) [43], to correct for B_0_ inhomogeneities and CEST imaging were performed based on the same RARE-based sequence proposed by Villano et al [44], which was adjusted to be compatible with this scanner and software version. WASSR was performed using 30 offsets from +1.5 ppm to -1.5 ppm, steps 0.1 ppm, TR 3,313 ms, TE 4.3 ms, spatial resolution 0.234 × 0.234 mm^2^, slice thickness 1 mm, matrix size 128 × 128, field-of-view 30 × 30 mm^2^, saturation pulse strength 0.2 $\mu$T, TA 2 min.

CEST images were obtained with following parameters: 120 frequency offsets from +6 ppm to -6 ppm, steps 0.1 ppm, TR 3,000 ms, TE 4.3 ms, spatial resolution: 0.234 × 0.234 mm^2^, slice thickness 1 mm, field-of-view 30 × 30 mm^2^, matrix size 128 × 128, saturation pulse strength 3 $\mu$T, saturation pulse length 3,000 ms, TA 7 min. WASSR and CEST imaging were done at baseline for APTw-CEST and repeated 24 min after 2-DG application *in vivo*. *In vitro*, a single WASSR and CEST acquisition was conducted. Then, a T1-weighted map was acquired using a RARE sequence to correct T1 times in the AREX metric as described in Eq. 7. The T1-weighted map was acquired using following parameters: variable TR 867–6,000 ms, TE 28 ms, spatial resolution 0.234 × 0.234 mm^2^, slice thickness 1 mm, matrix size 128 × 128, field-of-view 30 × 30 mm^2^, TA 4:27 min:s.

### CEST-MRI metrics: MTR_asym_, Lorentz amplitude, MTR_REX_ and AREX

The CEST z-spectrum (Eq. 1) is defined by the fraction of steady state magnetization before ${M}_{Z}^{0}\left(\Delta \omega \right)$ and after ${M}_{Z}\left(\Delta \omega \right)$ application of the radiofrequency pulse band. The commonly used MTR_asym_ is calculated by subtracting the CEST ratio (Eq. 1) on the y-axis of the z-spectrum for various frequency offsets $(\Delta \omega )$ on the x-axis, taken from opposite sides of the bulk water reference peak at 0 ppm (Eq. 2). 2-DG exhibits four exchange pools at +0.66 ppm, +1.28 ppm, +2.08 ppm and +2.88 ppm, with the most prominent exchange occurring at +1.28 ppm. This frequency is used as the 2-DG reference frequency offset [20]. For imaging of endogenous APTw-CEST signal, the reference frequency offset of +3.50 ppm was used.

1 $$Z\left(\Delta \omega \right)=\frac{{M}_{Z}\left(\Delta \omega \right)}{{M}_{Z}^{0}\left(\Delta \omega \right)}$$

2 $${MT}{R}_{{asym}}\left(\Delta \omega \right)=Z\left(-\Delta \omega \right)-Z\left(\Delta \omega \right)$$

The MTR_asym_, however, is sensitive to artifacts caused by motion, B_0_ and B_1_ inhomogeneities and the interference from other exchange pools such as amide, amine, the semi-solid magnetization transfer and NOE [45]. To mitigate these influences, advanced corrections based on Lorentzian fitting (Eq. 3) as proposed by Windschuh et al [46] can be applied post-acquisition. Here $I$ is the image intensity, ${I}_{0}$ the image intensity without pre-saturation, $K$ the Number of Lorentzian pools (in this case $K$ = 5), *ω* the frequency, ${a}_{k}$ the amplitude, ${\omega }_{k}^{c}$ the width and ${\sigma }_{k}$ the frequency offset of the Lorentzian fit.

3 $$L\left({a}_{k},{\omega }_{k}^{c},{\sigma }_{k}\right)=1-\frac{I}{{I}_{0}}={\sum }_{k=1}^{K}\frac{{a}_{k}}{1+4{\left(\frac{\omega -{\omega }_{k}^{c}}{{\sigma }_{k}}\right)}^{2}}$$

This is already established in APTw-CEST postprocessing but not common practice for the analysis of glucoCEST datasets. The z-spectrum is affected by spillover from adjacent pools. To address this, the MTR_REX_ metric accounts for these effects by subtracting the inverse sum of all other exchange pools [31]. ${Z}_{{lab}}$ represents the $Z$ value at the label frequency and is calculated in Eq. 4 by subtracting the sum over all Lorentzian fits from the parameter ${Z}_{{base}}$ which corrects the constant signal reduction. In Eq. 5, the reference $Z$ signal ${Z}_{{ref},j}$ is calculated by excluding the exchange pool $i$ at the label frequency. The final MTR_REX_ is then defined by the inverse subtraction of those two $Z$ values, as seen in Eq. 6. This is proven to be essential in APTw-CEST and should also benefit 2D-glucoCEST quantification since the 2-DG exchange pools are closely spaced. For the MTR_REX_ metric in APTw-CEST, the amide frequency at +3.50 ppm is chosen as the label, while signals from water (0 ppm), NOE (-3.5 ppm), MT (-2 ppm), and amine (+2.2 ppm) are removed. In 2-DG calculations, the label frequency is set to the dominant exchange pool at +1.28 ppm, while contributions from water at 0 ppm, and other 2-DG pools at +0.66 ppm, +2.08 ppm and +2.88 ppm are subtracted (Supplementary Tables 1, 2).

4 $${Z}_{{lab}}\left(\Delta \omega \right)={Z}_{{base}}-{\sum }_{i}^{n=K}L_{i}(\Delta \omega )$$

5 $${Z}_{{ref},j}\left(\Delta \omega \right)={Z}_{{base}}-{\sum }_{i\ne j}^{n=K}L_{i}(\Delta \omega )$$

6 $${{MTR}}_{{REX}}\left({\delta }_{i}\right)=\frac{1}{{Z}_{{lab}}({\delta }_{i})}-\frac{1}{{Z}_{{ref},i}({\delta }_{i})}$$

Since CEST is also influenced by ${T1}_{{obs}}$ relaxation times, the AREX metric (Eq. 7) was introduced to correct MTR_REX_ values by accounting for T1-relaxation effects [31]:

7 $${AREX}\left({\delta }_{i}\right)=\frac{{{MTR}}_{{REX}}\left({\delta }_{i}\right)}{{T1}_{{obs}}}$$

**Fig. 1:**
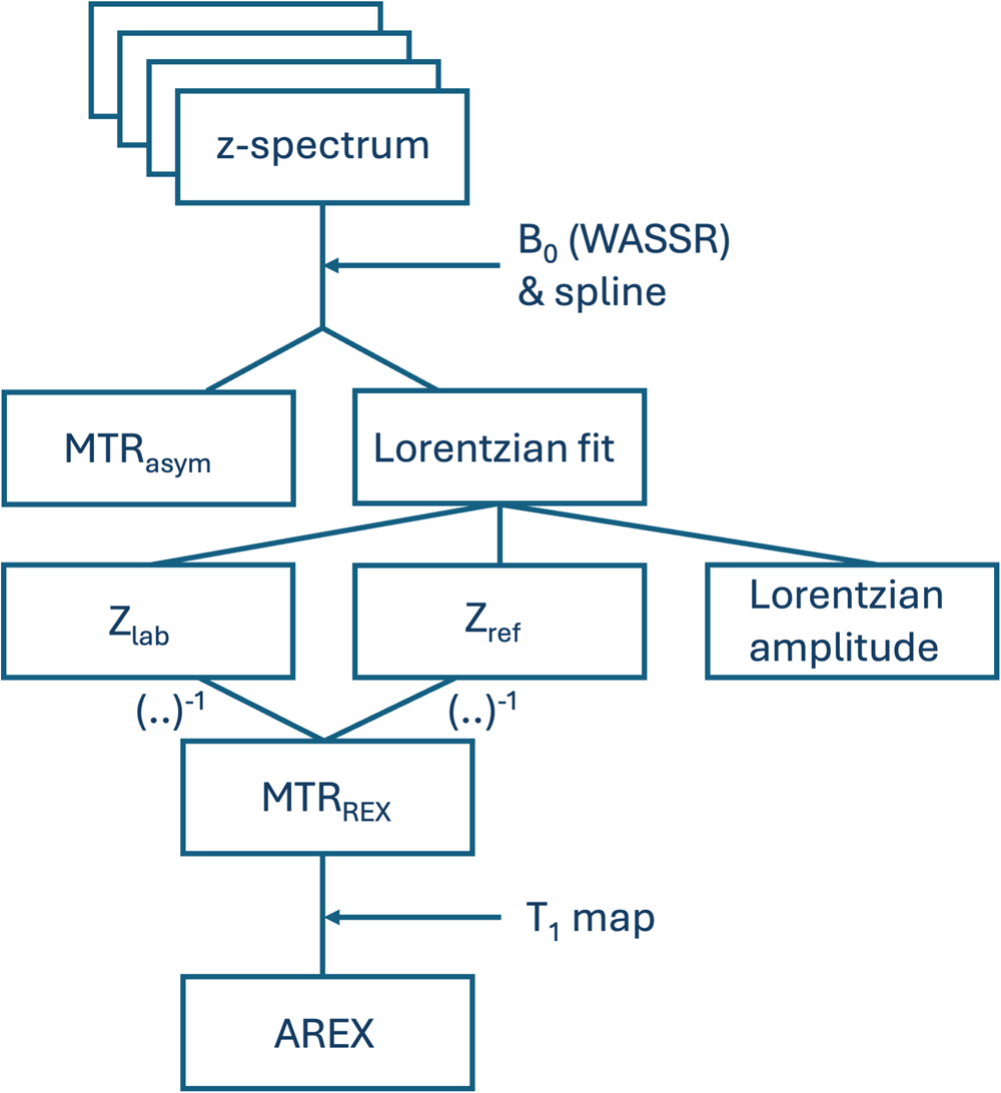
Scheme of postprocessing pipeline. After z-spectrum acquisition, B_0_ correction with WASSR and spline fitting is applied. Then the MTR_asym_ is computed and Lorentzian fitting is performed using two different 5-pool models according to APTw-CEST or 2D-glucoCEST (Supplementary Tables S1, S2). Simple amplitude extraction from Lorentzian fits allows the generation of parametric Lorentzian amplitude maps. Then, based on Lorentzian fitting parameters, Z_lab_ (Eq. 4) and Z_ref_ (Eq. 5) can be computed where the inverse sum results in the MTR_REX_ metric (Eq. 6). At the end, a T1 mapping allows for the computation of the T1-independent AREX metric (Eq. 7). AREX, Apparent exchange-dependent relaxation; MTR_asym_, Magnetization transfer ratio asymmetry; MTR_REX_, Magnetization transfer ratio relaxation exchange; WASSR, Water saturation shift referencing; Z_lab_, $Z$ value at the label frequency; Z_ref_, $Z$ value calculated by excluding the exchange pool $i$ at the label frequency

Finally, based on these four quantification metrics, delta maps were generated to assess signal differences before and after challenge application, as demonstrated in the case of 2-DG. The complete postprocessing pipeline is illustrated in Fig. 1.

### CEST postprocessing

For *in vitro* and *in vivo* CEST postprocessing, T2-weighted anatomical reference images (*.dcm), T1 maps (*.nii) and raw CEST data (*.2Dseq) were exported from ParaVision v360.33 software (Bruker BioSpin GmbH). The initial steps of CEST postprocessing included dataset import, B_0_ correction using WASSR images, spline fitting of z-spectra and Lorentzian fits. These steps were performed in MATLAB version R2023a (MathWorks) using previously published code (cest-sources.org) [47]. The remaining quantification was carried out using MATLAB code developed in-house. Image readings were performed by two supervised PhD students with experience in preclinical imaging, who consulted with each other during regions of interest (ROIs) placement to ensure consistency. Both readers were aware of the assignment of the animals to the respective groups. ROIs were manually drawn on axial T2-weighted anatomical reference images, and parametric CEST maps were generated according to Eqs. 2, 3, 6 and 7 and overlaid using the Fusion module of pmod (version 4.4, PMOD Technologies LLC, Switzerland) [48]. This process was repeated for egg white phantoms *in vitro* and *in vivo* pre-contrast images on endogenous APTw-CEST signals using a five-pool Lorentzian model (Supplementary Table 1) and for 2-DG phantoms *in vitro* and *in vivo* post-contrast images to assess exogenous 2D-glucoCEST, applying a different five-pool Lorentzian model to account for 2-DG exchange pools (Supplementary Table 2). In addition to inspection of the z-spectra *in vitro* for both APTw-CEST and 2D-glucoCEST, the CNR for the four quantification metrics was calculated based on a mean value within two signal producing ROIs $({{{\rm{\mu }}}}_{1/2})$ and their standard deviations $({{{\rm{\sigma }}}}_{1/2})$:

8 $${{\rm{CNR}}}=\frac{|{\mu }_{1}-{\mu }_{2}|}{\sqrt{{\sigma }_{1}^{2}+{\sigma }_{2}^{2}}}$$

### Statistical analysis

#### *In vitro* experiments

Coregistered maps and ROIs were exported from pmod as *.nii and evaluated with R version 4.4.0, 2024-04-24 using RStudio (Posit Software, PBC, Boston, USA). Values were reported as mean ± standard deviation. Additionally, CNR was calculated between consecutive concentrations according to Eq. 8.

#### *In vivo* experiments

Coregistered maps and ROIs were exported from pmod as *.nii, and statistical analysis was performed using R Studio. Maps with obvious artifacts (*e.g*., motion or stripe artifacts caused by the heating water bed) were excluded, and outliers were identified using Eq. 9.

9 $$Q3+1.5 \, \times \, {IQR} \, < \, x \, < \, Q1-1.5 \, \times \, {IQR}$$

where $Q1$ is the first quartile, $Q3$ is the third quartile, ${IQR}$ the interquartile range and $x$ the observed variable.

A flowchart of animal numbers and exclusions can be found in Supplementary Fig. S1. After all exclusions, the final sample sizes were as follows: APTw-CEST: *n* = 12 (Luminal A), *n* = 14 (HER2+) and *n* = 13 (triple-negative) and 2D-glucoCEST: *n* = 9 (Luminal A), *n* = 9 (HER2+), and *n* = 10 (triple-negative). Animals were assigned to experimental groups using random cage selection, ensuring that the average body weights per assigned group (~25 g) were the same to minimize baseline variability. A power calculation to determine sample sizes was conducted and can be found in the Supplementary Material. Tumor volumes were calculated from T2-weighted axial images using manual segmentation in PMOD based on voxel dimensions (Supplementary Table S3). All values are reported as mean ± standard deviation. Normality was assessed using the Shapiro–Wilk test. If the dataset showed normal distribution, one-way analysis of variance (ANOVA) was conducted with Tukey HSD *post hoc* pairwise comparisons. If the dataset was non-normally distributed, the Kruskal–Wallis test was performed with Benjamini–Hochberg *post hoc* corrections and pairwise comparisons. (*p < 0.05, **p < 0.01).

## Results

### *In vitro* experiments

**Fig. 2:**
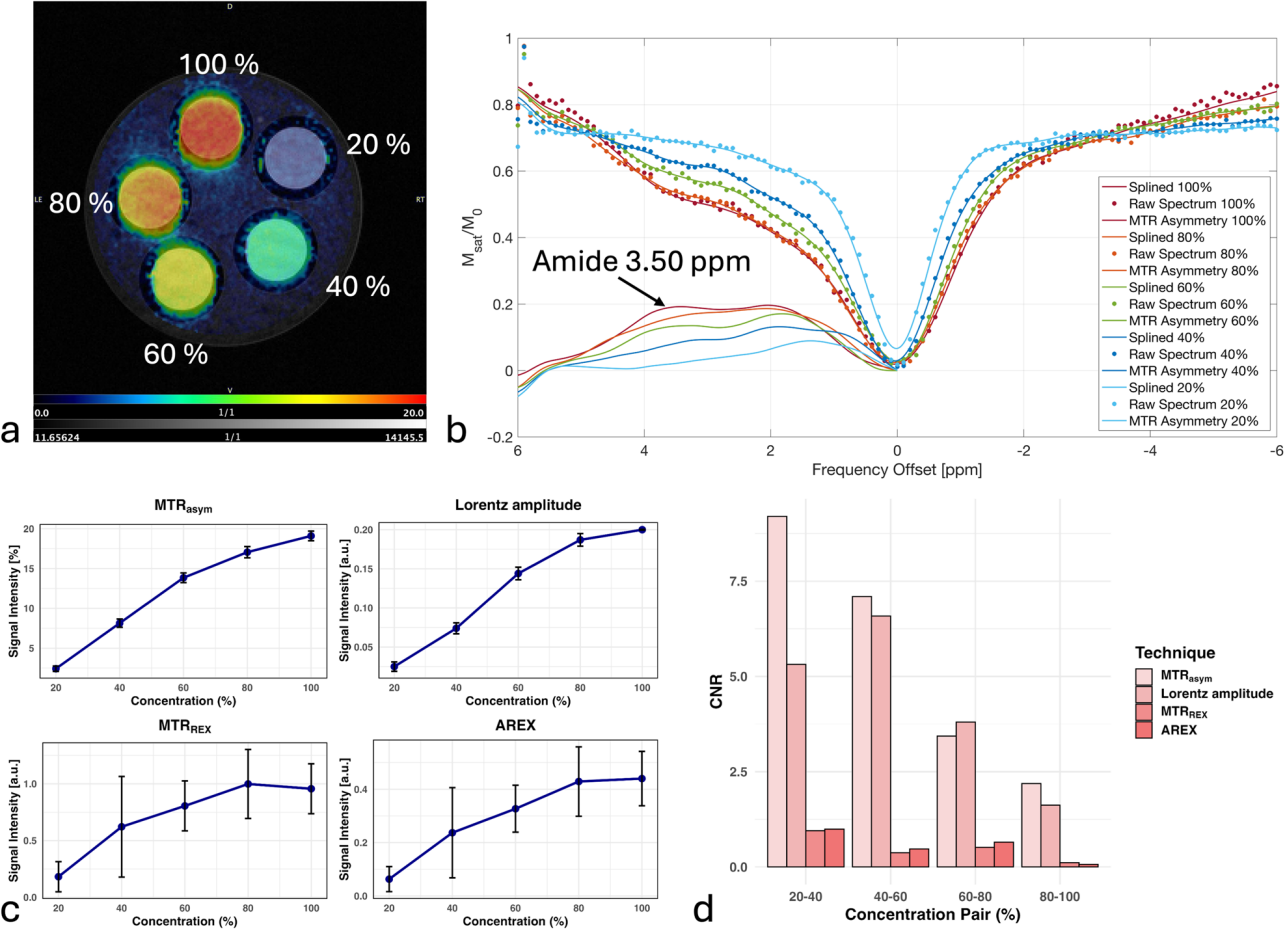
*In vitro* egg white phantoms to illustrate APTw-CEST contrast. **a** T2-weighted anatomical image overlayed with MTR_asym_ map of egg white + PBS phantoms at 5 different concentrations (20%, 40%, 60%, 80%, 100%). **b** Single-pixel z-spectra of egg white + PBS phantoms at 5 different concentrations. Dots represent raw data while solid lines show the spline fit. The MTR_asym_ can be seen in the lower left corner. The amide peak at +3.50 ppm is clearly visible and marked by an arrow. **c** Signal intensity of MTR_asym_, Lorentz amplitude, MTR_REX_ and AREX at 5 different concentrations. An increase in signal intensity with increasing concentration can be seen across all metrics. **d** CNR according to Eq. 8 between neighboring concentrations, highest CNR can be seen for the MTR_asym_, followed by Lorentzian amplitudes, AREX and MTR_REX_. AREX, Apparent exchange-dependent relaxation; CNR, Contrast-to-noise ratio; MTR_asym_, Magnetization transfer ratio asymmetry; MTR_REX_, Magnetization transfer ratio relaxation exchange

The CEST measurements demonstrated stable and reliable signal detection across different phantoms, allowing for clear peak identification and quantitative CNR comparisons. In egg white measurements, a stable APTw-CEST signal was observed, with a well-fitted spline and distinct amide peak at +3.50 ppm (Fig. 2b).

**Fig. 3:**
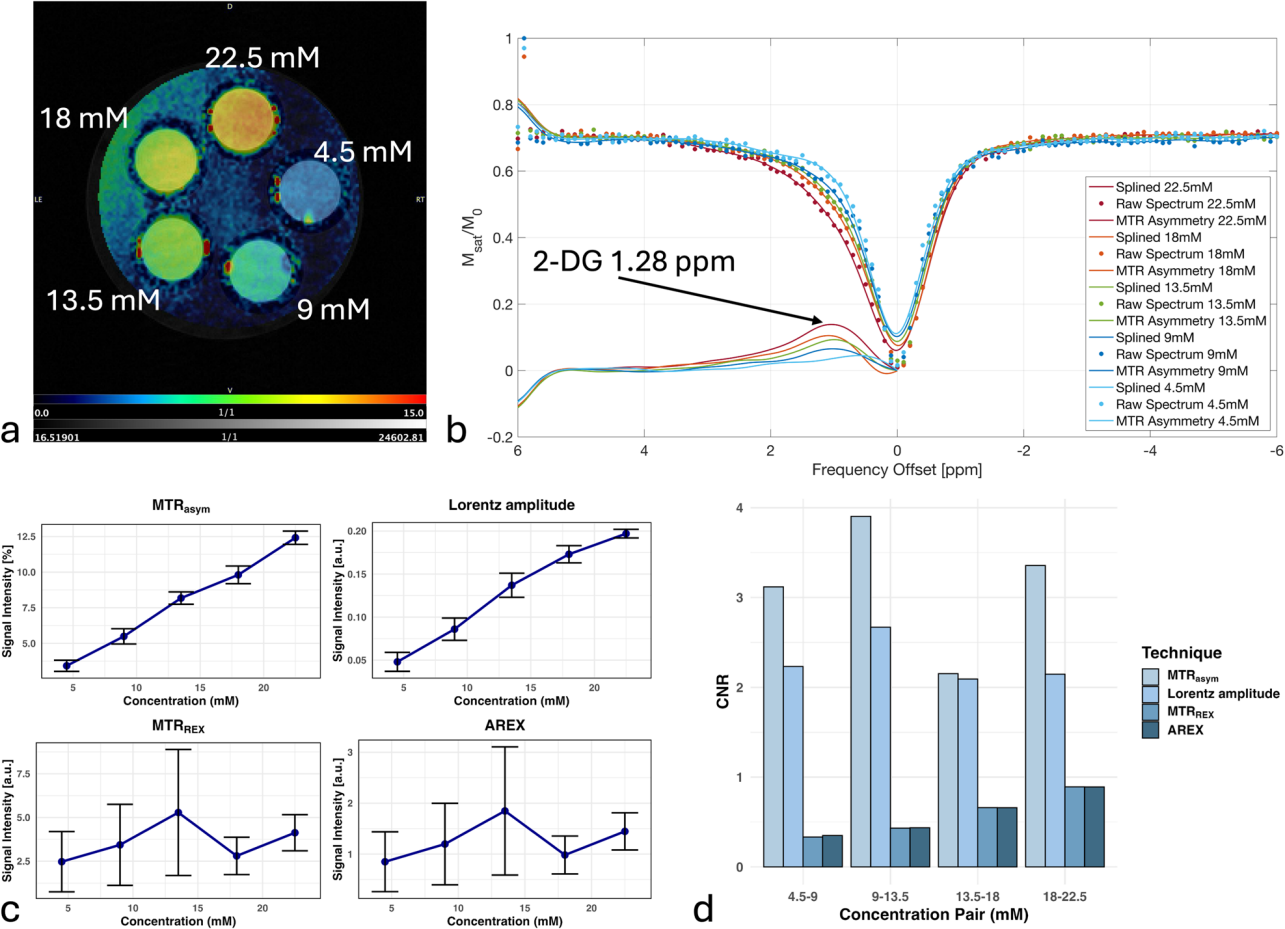
*In vitro* 2-DG phantoms to illustrate glucoCEST contrast. **a** T2-weighted anatomical image overlayed with MTR_asym_ map of a phantom containing 5 different concentrations of 2-DG in PBS (4.5 mM, 9 mM, 13.5 mM, 18 mM, 22.5 mM). **b** Single-pixel z-spectra of 2-DG + PBS phantoms. Dots represent raw data while solid lines show the spline fit. The MTR_asym_ can be seen in the lower left corner. The 2-DG peak at +1.28 ppm is clearly visible and marked by an arrow. **c** Signal intensity of MTR_asym_, Lorentz amplitude, MTR_REX_ and AREX at 5 different concentrations. An increase in signal intensity with increasing concentration can be seen across MTR_asym_ and Lorentz amplitude; however, for MTR_REX_ and AREX, the signal decreases for the two highest concentrations. **d** CNR according to Eq. 8 between neighboring concentrations, the highest CNR can be seen for the MTR_asym_, followed by Lorentzian amplitudes, AREX and MTR_REX_. 2-DG, 2-Deoxy-D-glucose; AREX, Apparent exchange-dependent relaxation; CNR, Contrast-to-noise ratio; MTR_asym_, Magnetization transfer ratio asymmetry; MTR_REX_, Magnetization transfer ratio relaxation exchange

For 2-DG phantoms at five different concentrations (4.5 mM, 9 mM, 13.5 mM, 18 mM, 22.5 mM), the 2D-glucoCEST z-spectrum remained stable with a well-fitted spline, and MTR_asym_ maxima increased with rising concentrations (Fig. 3b).

### *In vivo* experiments

**Fig. 4:**
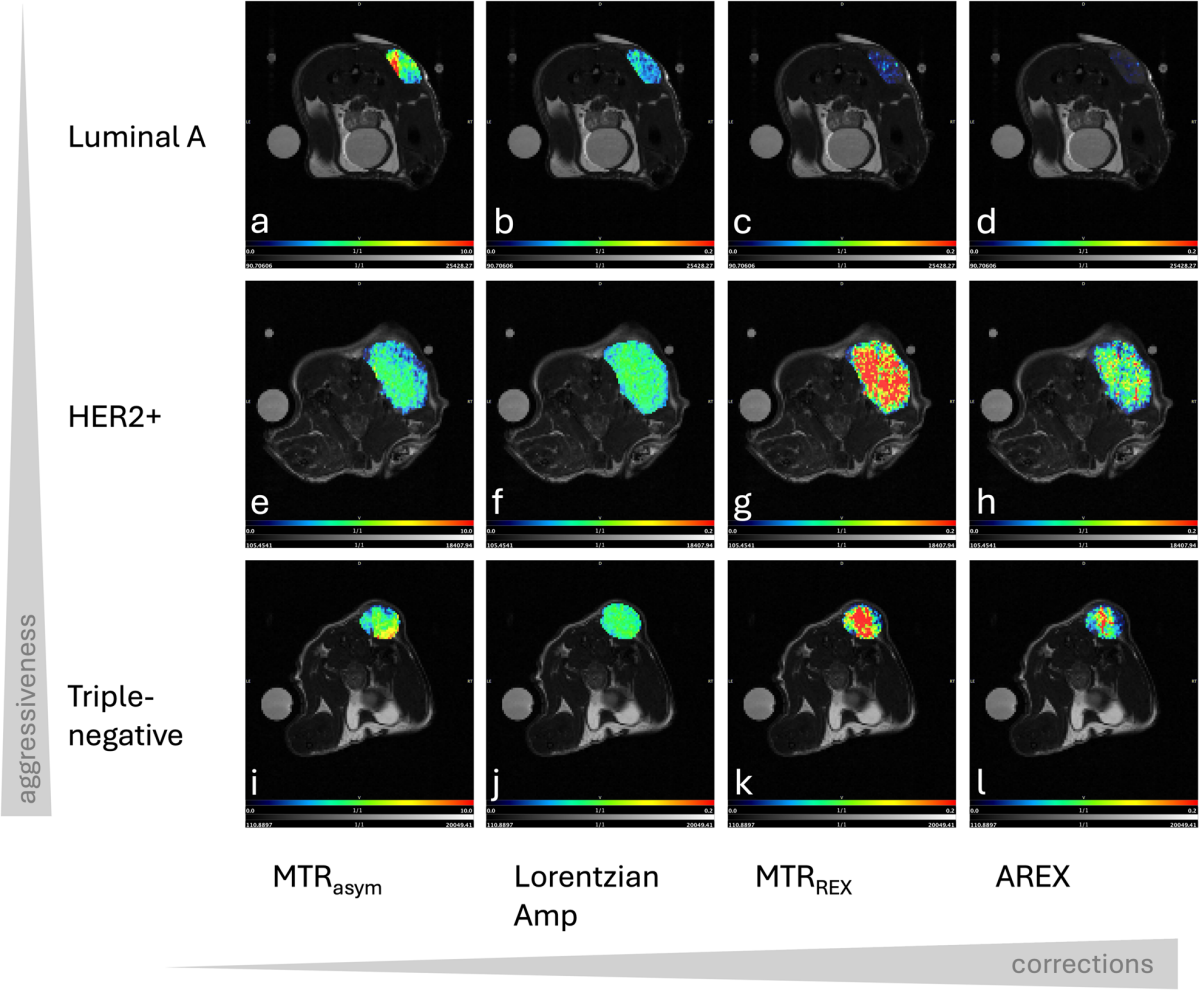
T2-weighted anatomical images in axial orientation with a phantom on the left and tumor at the dorsal side, overlapped with APTw-CEST parametric maps. The first row shows the Luminal A tumor, the second row the HER2+ tumor and the third row the triple-negative tumor. The following parametric maps are shown: (**a**, **e**, **i**) MTR_asym_, (**b**, **f**, **j**) Lorentz amplitude, (**c**, **g**, **k**) MTR_REX_ and (**d**, **h**, **l**) AREX. AREX, Apparent exchange-dependent relaxation; MTR_asym_, Magnetization transfer ratio asymmetry; MTR_REX_, Magnetization transfer ratio relaxation exchange

Advanced quantification metrics, including MTR_REX_ and AREX, revealed significant distinctions between BC subtypes for both APTw-CEST and 2D-glucoCEST. Distinct spatial variations across BC subtypes were most pronounced in the MTR_REX_ and AREX parametric maps for both APTw-CEST (Fig. 4) and 2D-glucoCEST images (Supplementary Fig. 2).

**Fig. 5:**
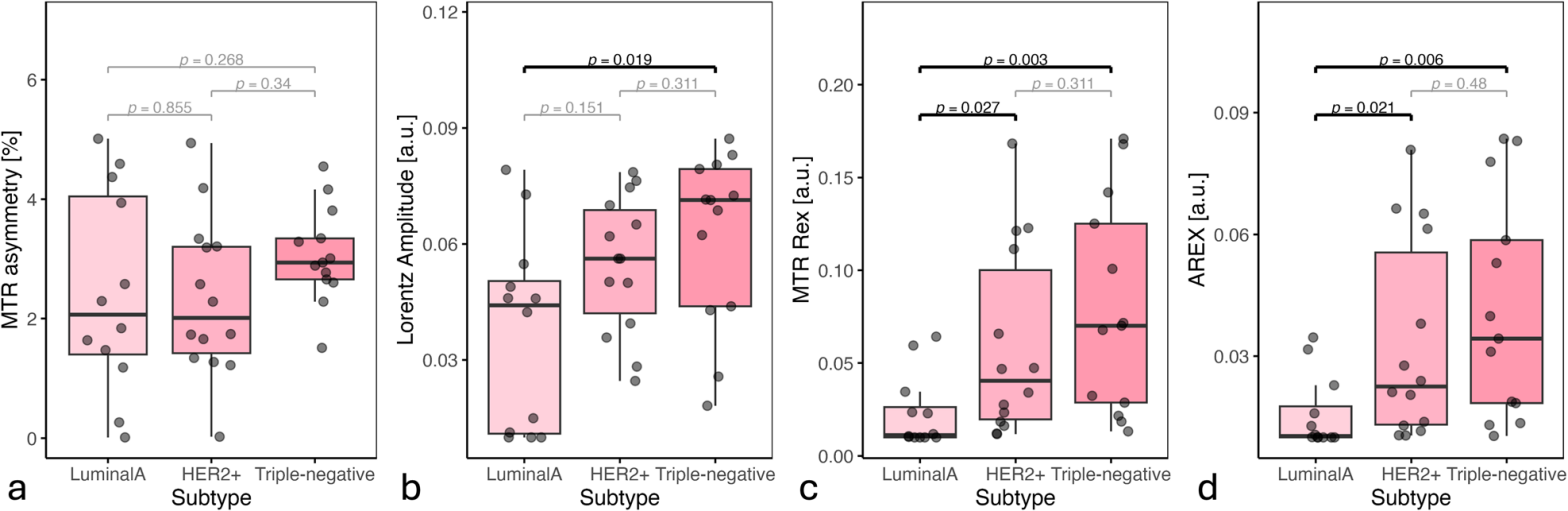
APTw-CEST signal across three different breast cancer subtypes. Light pink represents Luminal A tumors, medium pink HER2+ tumors and the dark pink represents triple-negative tumors, according to subtype aggressiveness. The boxplots show mean values over tumor ROIs for mice inoculated with the respective subtype. An increase in signal according to aggressiveness can be observed across all figures: (**a**) MTR_asym_, (**b**) Lorentz amplitudes, (**c**) MTR_REX_, and (**d**) AREX, where all metrics enable significant differentiation between the subtypes except for the MTR_asym_ in **a**. AREX, Apparent exchange-dependent relaxation; MTR_asym_, Magnetization transfer ratio asymmetry; MTR_REX_, Magnetization transfer ratio relaxation exchange

Among the four postprocessing metrics applied to APTw-CEST, AREX and MTR_REX_ (Fig. 5c, d) significantly differentiated Luminal A from HER2+ (*p = 0.021; *p = 0.027) and Luminal A from triple-negative (**p = 0.006; **p = 0.003), demonstrating rising APTw-CEST signals with increasing BC subtype aggressiveness. The Lorentzian amplitude (Fig. 5b) successfully differentiated only Luminal A from triple-negative (*p = 0.019). MTR_asym_ (Fig. 5a) was unable to distinguish between BC subtypes. Notably, none of the applied APTw-CEST metrics revealed significant differences between HER2+ and triple-negative tumors.

**Fig. 6:**
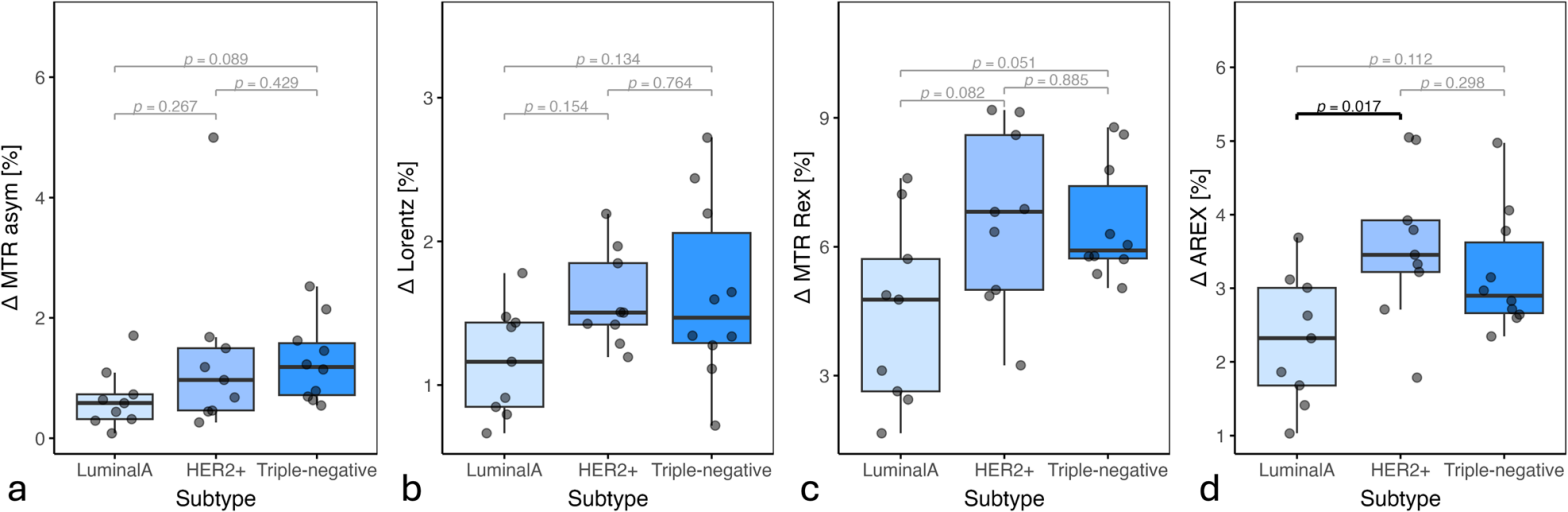
$\Delta$2D-glucoCEST signal across three different breast cancer subtypes. Light blue represents Luminal A tumors, medium blue HER2+ tumors and dark blue the triple-negative tumors according to subtype aggressiveness. The boxplots show mean values over tumor ROIs for mice inoculated with the respective subtype. A signal increase from Luminal A to HER2+ can be seen across all metrics: (**a**) MTR_asym_, (**b**) Lorentz amplitude, (**c**) MTR_REX_, and (**d**) significantly in AREX. However, only Lorentz amplitude, MTR_REX_ and AREX display a decrease in signal for the most aggressive triple-negative subtype. AREX, Apparent exchange-dependent relaxation; MTR_asym_, Magnetization transfer ratio asymmetry; MTR_REX_, Magnetization transfer ratio relaxation exchange

Among the four applied postprocessing metrics for 2D-glucoCEST, only $\Delta$AREX (Fig. 6d) was able to significantly differentiate Luminal A from HER2+ (*p = 0.017) and showed a decrease in the 2D-glucoCEST signal for the triple-negative subtype. $\Delta$MTR_REX_ (Fig. 6c), $\Delta$Lorentzian amplitudes (Fig. 6b) and $\Delta$MTR_asym_ (Fig. 6a) varied between BC subtypes but without any significance.

Mean values $\pm$ standard deviation for APTw-CEST and $\Delta$2D-glucoCEST signals are presented in Table 1. These values demonstrated a consistent increase in mean APTw-CEST signals with increasing BC subtype aggressiveness for the three postprocessing metrics that produced significant results (Lorentzian amplitude, MTR_REX_, AREX), whereas MTR_asym_ remains inconclusive. For $\Delta$2D-glucoCEST, $\Delta$AREX had a mean signal intensity of 2.3‒3.2% while $\Delta$MTR_REX_ had a mean signal intensity of 4.5‒6.5% as it is still influenced by T1. $\Delta$Lorentz amplitude and $\Delta$MTR_asym_ showed relatively low values of 0.7‒1.6%.

**Table 1:** Mean values (± standard deviation) for each quantification metric across the three breast cancer subtype groups for both APTw-CEST and $\Delta$2D-glucoCEST

APTw-CEST				
	MTR_asym_ (%)	Lorentz amplitude (a.u.)	MTR_REX_ (a.u.)	AREX (a.u.)
Luminal A	2.43 $\pm$ 1.69	0.037 $\pm$ 0.025	0.023 $\pm$0.020	0.016 $\pm$0.009
HER2+	2.34 ± 1.32	0.055 ± 0.018	0.059 ± 0.051	0.033 ± 0.025
Triple-negative	3.06 ± 0.80	0.062 ± 0.022	0.079 ± 0.057	0.041 ± 0.027

$\Delta$2D-glucoCEST				
	$\Delta$MTR_asym_ (%)	$\Delta$Lorentz amplitude (a.u.)	$\Delta$MTR_REX_ (a.u.)	$\Delta$AREX (a.u.)
Luminal A	0.653 ± 0.490	1.16 $\pm$0.38	4.45 ± 2.13	2.31 ± 0.88
HER2+	1.35 ± 1.45	1.59 ± 0.33	6.67 ± 2.07	3.59 ± 1.04
Triple-negative	1.28 ± 0.67	1.64 ± 0.63	6.52 ± 1.36	3.21 ± 0.82

Interquartile ranges can be found in Supplementary Table S4 and reflect a consistent distribution of values for all four metrics across tumors within each subtype

*2D* 2-Deoxy-D-glucose, *APTw* Amide proton transfer weighted, *AREX* Apparent exchange-dependent relaxation, *CEST* Chemical exchange saturation transfer, *MTR*_*asym*_ Magnetization transfer ratio asymmetry, *MTR*_*REX*_ Magnetization transfer ratio relaxation exchange

## Discussion

Metabolic CEST-MRI is a promising noninvasive imaging technique [7–10]. Particularly, the endogenous APTw-CEST [11–16, 49] and exogenous glucoCEST [17–23] are of importance as they provide insights into cancer biology. However, CEST imaging and quantification are influenced by various parameters related to hardware, imaging sequences, and postprocessing metrics. Recent efforts have aimed to address potential sources of artifacts, including field inhomogeneities, motion, and signal contamination from overlapping pools, through increasingly sophisticated postprocessing approaches [12, 13, 29–31, 43, 46, 50–52]. Early studies highlighted critical challenges, particularly the influence of the NOE on the MTR_asym_ of amide at +3.50 ppm.

This spectral interference complicates conventional quantification from mobile macromolecular relaxation processes occurring symmetrically opposite the amide peak in the z-spectrum at around -3.50 ppm upfield from the water resonance. Furthermore, fat correction was challenging [15]. To overcome these technical hurdles, advanced postprocessing metrics were developed. Among them, MTR_REX_ and AREX have demonstrated superior performance in glioblastoma and ischemic stroke studies [27, 28, 31, 46], allowing a differentiation between healthy and pathological brain tissue.

To our knowledge, this is one of the first studies to apply these postprocessing metrics in xenograft models of BCs, where we anticipate similarly promising results as in glioblastoma studies, as the underlying biological processes—specifically protein metabolism, perfusion, and pH regulation—are known to be similar between glioblastoma and BC tissues [10].

Among the four postprocessing metrics applied, only MTR_REX_ and AREX allow molecular subtyping with APTw-CEST *in vivo*. On the other hand, in 2D-glucoCEST only AREX shows similar results. Notably, in our *in vitro* experiments, APTw-CEST spectra from egg white were stable with increasing protein concentration across all metrics, with MTR_asym_ yielding the highest CNR. In 2-DG phantoms, slight deviations in MTR_REX_ and AREX curves at the highest concentrations coincided with minor image artifacts—likely due to complex phantom preparation. These findings indicated that CEST quantification varies depending on the contrast type, with different signal intensity trends observed with rising concentrations for APTw-CEST and 2D-glucoCEST phantom signals.

Although few studies have assessed the clinical utility of APTw-CEST in BC, a recent review of 14 studies reported contradictory findings on whether the APTw signals are higher or lower in malignant *versus* benign breast lesions [32]. For instance, one study reported an APTw signal of 2.7% in benign and 1.2% in malignant lesions [33], whereas most reported elevated malignant values, such as 1.55% *versus* 0.54% in Liu et al [34].

BC subtype-specific results from Kamitani et al showed 1.74% (Luminal type), 1.83% (HER2+) and 2.75% (triple-negative) [36]. In line with this, Zhang et al [35] reported mean APTw signals of 2.30% (Luminal A), 2.30% (Luminal B), 2.23% (HER2+) and 3.20% (triple-negative) in a prospective cohort of 205 patients, further underlining the clinical relevance of CEST imaging. While their work compared different modalities such as dynamic contrast-enhanced (DCE) imaging, intravoxel incoherent motion (IVIM) and CEST, our study adds value by systematically identifying the most suitable CEST postprocessing metric for reliable molecular subtyping, which can guide future translational and clinical studies.

While most studies were conducted at 3 T, our 9.4-T preclinical data followed the same trend of increasing APTw signal with subtype aggressiveness, supporting the majority of clinical findings. Thus, our findings hold translational relevance for clinical scanners operating at 3 T, where APTw-CEST has already demonstrated feasibility by keeping efficient acquisition times between 4 and 7 min in several clinical studies [33–35].

Notably, all of the mentioned studies relied solely on the MTR_asym_ metric, except for Zhang et al [14], who additionally incorporated Lorentzian amplitude analysis. However, it has been widely recognized that the specificity of the MTR_asym_ is limited, as it is influenced by other exchange pools. To overcome these limitations, our study applied multi-pool CEST contrast using MTR_REX_, while diffusion-related effects were accounted for by calculating AREX, thereby enhancing both specificity and biological interpretability.

Our findings show that while Lorentzian fitting modestly improved contrast, only corrections employing advanced postprocessing metrics, such as MTR_REX_ and AREX, allowed accurate molecular subtyping, enabling differentiation of less aggressive Luminal A from moderate aggressive HER2+ and highly aggressive triple-negative BCs. Future studies should investigate the integration of these postprocessing metrics into clinical breast MRI, as additional validation, standardization, and multi-center evaluation will be necessary before their potential to enhance diagnostic utility and reproducibility can be fully realized.

GlucoCEST allows quantification of glucose metabolism typically elevated in cancer tissue and has emerged as a potential complementary technique to [^18^F]FDG-PET. In contrast to APTw-CEST, advanced postprocessing metrics have not yet been widely applied to glucoCEST imaging. This is the first study quantifying a 2D-glucoCEST dataset beyond MTR_asym_ by incorporating Lorentzian amplitudes, MTR_REX_ and AREX analysis. In a study by Anemone et al, an MTR_asym_ signal of 1‒2% at 3 T and 2‒3% at 7 T was observed in a melanoma mouse model for two different glucose analogs, similar to what we found [53]. Our results demonstrate, however, that only AREX enables molecular subtyping in BC with increasing 2D-glucoCEST signals from the Luminal A to the more aggressive HER2 + BC subtype. However, the triple-negative BC subtype displayed an unexpectedly lower signal compared to the HER2+ subtype. This contradictory effect may be attributed to two factors: (1) insufficient delivery of 2-DG, particularly to the tumor core, due to leaky vasculature from rapid tumor growth [54], and (2) an increased intracellular pH in tumor cells, which could shift the base-catalyzed CEST from a slow-to-intermediate to a fast exchange rate, leading to a reduction in its signal due to undetectably fast exchange [55–57]. This is consistent with findings by Chan et al [22], who similarly reported a reduced glucoCEST contrast in the more aggressive triple-negative subtype. They suggested, this may be due to reduced perfusion, less favorable extracellular pH, or diminished glucose retention in highly aggressive tumors. Future studies could complement CEST-MRI with dynamic contrast-enhanced (DCE) MRI to noninvasively assess vascular perfusion and permeability *in vivo* and would provide functional information directly relevant to the proposed mechanism, while vascular immunohistochemistry could additionally validate these findings.

While glucoCEST holds promise as a noninvasive metabolic imaging technique, its clinical translation faces several barriers. These include rapid wash-out of glucose post-injection, spectral overlap between hydroxyl exchange and water resonance—specifically at low field strengths—and vascular integrity [20]. Although alternative glucose analogs like 3-O-methyl-D-glucose, D-glucosamine, and others have been proposed to address some of these limitations [20, 53, 58], their hydroxyl exchange sites remain close to the water resonance. Therefore, improvements in glucoCEST performance at clinical field strengths may be limited with these analogs alone. However, as seen in our study, even when applying an advanced postprocessing metric using AREX, 2D-glucoCEST remained less effective than APTw-CEST in differentiating BC subtypes.

The study is hampered by some limitations. First, magnetic field shimming during CEST acquisition affected image quality, leading to the exclusion of some datasets. Second, while B_0_ inhomogeneities were corrected using WASSR, B_1_ inhomogeneities were not addressed, as methods such as WASABI [52] could not be implemented due to time constraints. Incorporating B_1_ corrections may further improve quantification accuracy.

Additionally, we acknowledge that tumor sizes differed by up to a factor of three between Luminal A and the more aggressive HER2+ and triple-negative BC subtypes, although cross-sectional areas remained largely comparable (3.8 ± 2.2 mm^2^, mean ± standard deviation), and therefore, a systematic bias from tumor size differences is unlikely. Moreover, although three major and biologically distinct subtypes (Luminal A, HER2+, triple-negative BC) were included, Luminal B could not be feasibly added. Furthermore, interobserver variability was not assessed, and measurements were performed by non-radiologists.

Despite these limitations, our study is strengthened by what constitutes a comparatively large and diverse dataset for a preclinical xenograft study, encompassing three biologically distinct and clinically relevant molecular BC subtypes. While this cannot be directly compared in scale to clinical cohorts such as Zhang et al [35] with 205 participants, it nevertheless provides an important experimental foundation and lays the groundwork for future validation in additional tumor models and clinical research.

In conclusion, our study highlights the importance of advanced postprocessing metrics in APTw-CEST and 2D-glucoCEST imaging. The accuracy of molecular subtyping in a BC model varies considerably depending on the postprocessing metric applied. Among the four evaluated metrics, MTR_REX_ and AREX emerged as the most effective for both APTw-CEST and 2D-glucoCEST. Nevertheless, the advantages of advanced postprocessing metrics are more pronounced in APTw-CEST compared to 2D-glucoCEST. From a clinical perspective, these findings are highly relevant, as they pave the way for noninvasive, imaging-based biomarkers for improved cancer characterization.

## Supplementary information

ELECTRONIC SUPPLEMENTARY MATERIAL

Supplementary Tables

## Data Availability

The datasets used and/or analyzed during the current study are available from the corresponding author on reasonable request.

## Abbreviations

[^18^F]FDG: [^18^F]Fluorodeoxyglucose
2-DG: 2-deoxy-D-glucose
APTw: Amide proton transfer-weighted
AREX: Apparent exchange-dependent relaxation
BC: Breast cancer
CEST: Chemical exchange saturation transfer
CNR: Contrast-to-noise ratio
HER2: Human epidermal growth factor receptor 2
IQR: Interquartile range
MRI: Magnetic resonance imaging
MTR_asym_: Magnetization transfer ratio asymmetry
MTR_REX_: Magnetization transfer ratio relaxation exchange
NOE: Nuclear Overhauser effect
PBS: Phosphate-buffered saline
PET: Positron emission tomography
PR: Progesterone receptor
RARE: Rapid acquisition with relaxation enhancement
ROI: Region of interest
TA: Acquisition time
TE: Echo time
TR: Repetition time
WASSR: Water saturation shift referencing

## References

[1] van Zijl PCM, Yadav NN (2011) Chemical exchange saturation transfer (CEST): what is in a name and what isn’t? Magn Reson Med 65:927–948. 10.1002/mrm.22761 21337419 10.1002/mrm.22761 PMC3148076

[2] Wolff SD, Balaban RS (1989) Magnetization transfer contrast (MTC) and tissue water proton relaxation in vivo. Magn Reson Med 10:135–144. 10.1002/mrm.1910100113 2547135 10.1002/mrm.1910100113

[3] Zhou J, van Zijl PCM (2006) Chemical exchange saturation transfer imaging and spectroscopy. Prog Nucl Magn Reson Spectrosc 48:109–136. 10.1016/j.pnmrs.2006.01.001 10.1016/j.pnmrs.2020.06.001 33198968

[4] Sherry AD, Woods M (2008) Chemical exchange saturation transfer contrast agents for magnetic resonance imaging. Annu Rev Biomed Eng 10:391–411. 10.1146/annurev.bioeng.9.060906.151929 18647117 10.1146/annurev.bioeng.9.060906.151929 PMC2709739

[5] Ward KM, Aletras AH, Balaban RS (2000) A new class of contrast agents for MRI based on proton chemical exchange dependent saturation transfer (CEST). J Magn Reson 143:79–87. 10.1006/jmre.1999.1956 10698648 10.1006/jmre.1999.1956

[6] Vinogradov E, Sherry AD, Lenkinski RE (2013) CEST: from basic principles to applications, challenges and opportunities. J Magn Reson 229:155–172. 10.1016/j.jmr.2012.11.024 23273841 10.1016/j.jmr.2012.11.024 PMC3602140

[7] Consolino L, Anemone A, Capozza M et al (2020) Non-invasive investigation of tumor metabolism and acidosis by MRI-CEST imaging. Front Oncol 10:161. 10.3389/fonc.2020.00161 32133295 10.3389/fonc.2020.00161 PMC7040491

[8] Goldenberg JM, Pagel MD (2019) Assessments of tumor metabolism with CEST MRI. NMR Biomed 32:e3943. 10.1002/nbm.3943 29938857 10.1002/nbm.3943 PMC7377947

[9] Prinz D, Bartsch SJ, Ehret V, Friske J, Pinker K, Helbich TH (2024) Multiparametrische magnetresonanztomographie der brust. Radiologie 65:162–169. 10.1007/s00117-024-01390-1 39611894 10.1007/s00117-024-01390-1 PMC11845421

[10] Gao T, Zou C, Li Y, Jiang Z, Tang X, Song X (2021) A brief history and future prospects of CEST MRI in clinical non-brain tumor imaging. Int J Mol Sci 22:11559. 10.3390/ijms222111559 34768990 10.3390/ijms222111559 PMC8584005

[11] Zhou J, Lal B, Wilson DA, Laterra J, van Zijl PCM (2003) Amide proton transfer (APT) contrast for imaging of brain tumors. Magn Reson Med 50:1120–1126. 10.1002/mrm.10651 14648559 10.1002/mrm.10651

[12] Khlebnikov V, Polders D, Hendrikse J et al (2017) Amide proton transfer (APT) imaging of brain tumors at 7 T: the role of tissue water T1-relaxation properties. Magn Reson Med 77:1525–1532. 10.1002/mrm.26232 27060863 10.1002/mrm.26232

[13] Xu J, Zaiss M, Zu Z et al (2014) On the origins of chemical exchange saturation transfer (CEST) contrast in tumors at 9.4T. NMR Biomed 27:406–416. 10.1002/nbm.3075 24474497 10.1002/nbm.3075 PMC3972041

[14] Zhang S, Rauch GM, Adrada BE et al (2021) Assessment of early response to neoadjuvant systemic therapy in triple-negative breast cancer using amide proton transfer–weighted chemical exchange saturation transfer MRI: a pilot study. Radiol Imaging Cancer 3:e200155. 10.1148/rycan.2021200155 34477453 10.1148/rycan.2021200155 PMC8489465

[15] Zimmermann F, Korzowski A, Breitling J et al (2020) A novel normalization for amide proton transfer CEST MRI to correct for fat signal–induced artifacts: application to human breast cancer imaging. Magn Reson Med 83:920–934. 10.1002/mrm.27983 31532006 10.1002/mrm.27983

[16] Zhou J, Zaiss M, Knutsson L et al (2022) Review and consensus recommendations on clinical APT-weighted imaging approaches at 3T: application to brain tumors. Magn Reson Med 88:546–574. 10.1002/mrm.29241 35452155 10.1002/mrm.29241 PMC9321891

[17] Kim M, Eleftheriou A, Ravotto L et al (2022) What do we know about dynamic glucose-enhanced (DGE) MRI and how close is it to the clinics? Horizon 2020 GLINT consortium report. MAGMA 35:87–104. 10.1007/s10334-021-00994-1 35032288 10.1007/s10334-021-00994-1 PMC8901523

[18] Walker-Samuel S, Ramasawmy R, Torrealdea F et al (2013) In vivo imaging of glucose uptake and metabolism in tumors. Nat Med 19:1067–1072. 10.1038/nm.3252 23832090 10.1038/nm.3252 PMC5275770

[19] Rivlin M, Horev J, Tsarfaty I, Navon G (2013) Molecular imaging of tumors and metastases using chemical exchange saturation transfer (CEST) MRI. Sci Rep 3:3045. 10.1038/srep03045 24157711 10.1038/srep03045 PMC7365327

[20] Knutsson L, Xu X, Van Zijl PCM, Chan KWY (2023) Imaging of sugar‐based contrast agents using their hydroxyl proton exchange properties. NMR Biomed 36:e4784. 10.1002/nbm.4784 35665547 10.1002/nbm.4784 PMC9719573

[21] Capozza M, Anemone A, Dhakan C et al (2022) GlucoCEST MRI for the evaluation response to chemotherapeutic and metabolic treatments in a murine triple-negative breast cancer: a comparison with [18F]F-FDG-PET. Mol Imaging Biol 24:126–134. 10.1007/s11307-021-01637-6 34383241 10.1007/s11307-021-01637-6

[22] Chan KWY, McMahon MT, Kato Y et al (2012) Natural D-glucose as a biodegradable MRI contrast agent for detecting cancer. Magn Reson Med 68:1764–1773. 10.1002/mrm.24520 23074027 10.1002/mrm.24520 PMC3505108

[23] Nasrallah FA, Pagès G, Kuchel PW, Golay X, Chuang K-H (2013) Imaging brain deoxyglucose uptake and metabolism by glucocest MRI. J Cereb Blood Flow Metab 33:1270–1278. 10.1038/jcbfm.2013.79 23673434 10.1038/jcbfm.2013.79 PMC3734779

[24] Zaiss M, Herz K, Deshmane A et al (2019) Possible artifacts in dynamic CEST MRI due to motion and field alterations. J Magn Reson 298:16–22. 10.1016/j.jmr.2018.11.002 30500568 10.1016/j.jmr.2018.11.002

[25] Foo LS, Yap W-S, Hum YC, Manan HA, Tee YK (2020) Analysis of model-based and model-free CEST effect quantification methods for different medical applications. J Magn Reson 310:106648. 10.1016/j.jmr.2019.106648 31760147 10.1016/j.jmr.2019.106648

[26] Zhang X-Y, Wang F, Li H et al (2017) CEST imaging of fast exchanging amine pools with corrections for competing effects at 9.4 T. NMR Biomed 30:e3715. 10.1002/nbm.3715 10.1002/nbm.3715 PMC5490838 28272785

[27] Steidl E, Neuhaus E, Shrestha M et al (2025) Pathological tissue changes in brain tumors affect the pH-sensitivity of the T1-corrected apparent exchange dependent relaxation (AREX) of the amide protons. NMR Biomed 38:e5285. 10.1002/nbm.5285 39467029 10.1002/nbm.5285 PMC11602268

[28] Zaiss M, Windschuh J, Paech D et al (2015) Relaxation-compensated CEST-MRI of the human brain at 7 T: unbiased insight into NOE and amide signal changes in human glioblastoma. Neuroimage 112:180–188. 10.1016/j.neuroimage.2015.02.040 25727379 10.1016/j.neuroimage.2015.02.040

[29] Zhou IY, Wang E, Cheung JS, Zhang X, Fulci G, Sun PZ (2017) Quantitative chemical exchange saturation transfer (CEST) MRI of glioma using image downsampling expedited adaptive least-squares (IDEAL) fitting. Sci Rep 7:84. 10.1038/s41598-017-00167-y 28273886 10.1038/s41598-017-00167-y PMC5427899

[30] Heo H-Y, Lee D-H, Zhang Y et al (2017) Insight into the quantitative metrics of chemical exchange saturation transfer (CEST) imaging. Magn Reson Med 77:1853–1865. 10.1002/mrm.26264 27170222 10.1002/mrm.26264 PMC5107181

[31] Zaiss M, Xu J, Goerke S et al (2014) Inverse Z-spectrum analysis for spillover-, MT-, and T1-corrected steady-state pulsed CEST-MRI—application to pH-weighted MRI of acute stroke. NMR Biomed 27:240–252. 10.1002/nbm.3054 24395553 10.1002/nbm.3054 PMC4520220

[32] Lee RC, Boparai MS, Duong TQ (2025) Detection of breast cancer lesions using APT weighted MRI: a systematic review. J Transl Med 23:141. 10.1186/s12967-025-06153-7 39891260 10.1186/s12967-025-06153-7 PMC11786454

[33] Li Y, Zhang Y, Tian L et al (2024) 3D amide proton transfer-weighted imaging may be useful for diagnosing early-stage breast cancer: a prospective monocentric study. Eur Radiol Exp 8:41. 10.1186/s41747-024-00439-z 38584248 10.1186/s41747-024-00439-z PMC10999404

[34] Liu Z, Wen J, Wang M et al (2023) Breast amide proton transfer imaging at 3 T: diagnostic performance and association with pathologic characteristics. J Magn Reson Imaging 57:824–833. 10.1002/jmri.28335 35816177 10.1002/jmri.28335

[35] Zhang N, Shao X, Xu L et al (2025) Three-dimensional turbo-spin-echo amide proton transfer-weighted and intravoxel incoherent motion imaging MRI for triple-negative breast cancer: a comparison with molecular subtypes and histological grades. BMC Cancer 25:465. 10.1186/s12885-025-13879-6 40082810 10.1186/s12885-025-13879-6 PMC11907953

[36] Kamitani T, Sagiyama K, Yamasaki Y et al (2023) Amide proton transfer (APT) imaging of breast cancers and its correlation with biological status. Clin Imaging 96:38–43. 10.1016/j.clinimag.2023.02.002 36773531 10.1016/j.clinimag.2023.02.002

[37] Zaric O, Farr A, Poblador Rodriguez E et al (2019) 7T CEST MRI: a potential imaging tool for the assessment of tumor grade and cell proliferation in breast cancer. Magn Reson Imaging 59:77–87. 10.1016/j.mri.2019.03.004 30880110 10.1016/j.mri.2019.03.004

[38] Yu T, Li L, Shi J et al (2024) Predicting histopathological types and molecular subtype of breast tumors: a comparative study using amide proton transfer-weighted imaging, intravoxel incoherent motion and diffusion kurtosis imaging. Magn Reson Imaging 105:37–45. 10.1016/j.mri.2023.10.010 37890802 10.1016/j.mri.2023.10.010

[39] Cronin AE, Liebig P, Detombe SA, Duggal N, Bartha R (2024) Reproducibility of 3D pH-weighted chemical exchange saturation transfer contrast in the healthy cervical spinal cord. NMR Biomed 37:e5103. 10.1002/nbm.5103 38243648 10.1002/nbm.5103

[40] Percie du Sert N, Hurst V, Ahluwalia A et al (2020) The ARRIVE guidelines 2.0: updated guidelines for reporting animal research. J Cereb Blood Flow Metab 40:1769–1777. 10.1177/0271678X20943823 32663096 10.1177/0271678X20943823 PMC7430098

[41] Bartsch SJ, Brožová K, Fürböck C et al (2024) Methodological aspects of correlative, multimodal, multiparametric breast cancer imaging: from data acquisition to image processing for AI-based radioproteomics in a preclinical setting. Front Biomater Sci 3:1420114. 10.3389/fbiom.2024.1420114

[42] Dall G, Vieusseux J, Unsworth A, Anderson R, Britt K (2015) Low dose, low cost estradiol pellets can support MCF-7 tumour growth in nude mice without bladder symptoms. J Cancer 6:1331–1336. 10.7150/jca.10890 26640593 10.7150/jca.10890 PMC4643089

[43] Kim M, Gillen J, Landman BA, Zhou J, van Zijl PCM (2009) Water saturation shift referencing (WASSR) for chemical exchange saturation transfer (CEST) experiments. Magn Reson Med 61:1441–1450. 10.1002/mrm.21873 19358232 10.1002/mrm.21873 PMC2860191

[44] Villano D, Romdhane F, Irrera P et al (2021) A fast multislice sequence for 3D MRI-CEST pH imaging. Magn Reson Med 85:1335–1349. 10.1002/mrm.28516 33031591 10.1002/mrm.28516 PMC7756816

[45] Zaiss M, Bachert P (2013) Chemical exchange saturation transfer (CEST) and MR Z-spectroscopy in vivo: a review of theoretical approaches and methods. Phys Med Biol 58:R221. 10.1088/0031-9155/58/22/R221 24201125 10.1088/0031-9155/58/22/R221

[46] Windschuh J, Zaiss M, Meissner J-E et al (2015) Correction of B1-inhomogeneities for relaxation-compensated CEST imaging at 7 T. NMR Biomed 28:529–537. 10.1002/nbm.3283 25788155 10.1002/nbm.3283

[47] CEST sources (2014) CEST resources for chemical exchange saturation transfer MRI. Available via https://cest-sources.org/doku.php. Accessed 9 Dec 2024

[48] PMOD Technologies LLC (2023) PMOD: biomedical image quantification. Available via https://www.pmod.com/web/. Accessed 10 Jun 2025

[49] Jones CK, Schlosser MJ, van Zijl PCM, Pomper MG, Golay X, Zhou J (2006) Amide proton transfer imaging of human brain tumors at 3T. Magn Reson Med 56:585–592. 10.1002/mrm.20989 16892186 10.1002/mrm.20989

[50] Yan M, Bie C, Jia W, Liu C, He X, Song X (2024) Synthesis of higher-B0 CEST Z-spectra from lower-B0 data via deep learning and singular value decomposition. NMR Biomed 37:e5221. 10.1002/nbm.5221 39113170 10.1002/nbm.5221

[51] Huang J, Lai JHC, Tse K-H et al (2022) Deep neural network based CEST and AREX processing: application in imaging a model of Alzheimer’s disease at 3 T. Magn Reson Med 87:1529–1545. 10.1002/mrm.29044 34657318 10.1002/mrm.29044

[52] Schuenke P, Windschuh J, Roeloffs V, Ladd ME, Bachert P, Zaiss M (2017) Simultaneous mapping of water shift and B1 (WASABI)—application to field-Inhomogeneity correction of CEST MRI data. Magn Reson Med 77:571–580. 10.1002/mrm.26133 26857219 10.1002/mrm.26133

[53] Anemone A, Capozza M, Arena F et al (2021) In vitro and in vivo comparison of MRI chemical exchange saturation transfer (CEST) properties between native glucose and 3-O-methyl-D-glucose in a murine tumor model. NMR Biomed 34:e4602. 10.1002/nbm.4602 34423470 10.1002/nbm.4602 PMC9285575

[54] Yen T-H, Lee G-D, Chai J-W et al (2016) Characterization of a murine xenograft model for contrast agent development in breast lesion malignancy assessment. J Biomed Sci 23:46. 10.1186/s12929-016-0261-4 27188327 10.1186/s12929-016-0261-4 PMC4869355

[55] Liberti MV, Locasale JW (2016) The Warburg effect: how does it benefit cancer cells? Trends Biochem Sci 41:211–218. 10.1016/j.tibs.2015.12.001 26778478 10.1016/j.tibs.2015.12.001 PMC4783224

[56] McVicar N, Li AX, Gonçalves DF et al (2014) Quantitative tissue pH measurement during cerebral ischemia using amine and amide concentration-independent detection (AACID) with MRI. J Cereb Blood Flow Metab 34:690–698. 10.1038/jcbfm.2014.12 24496171 10.1038/jcbfm.2014.12 PMC3982091

[57] Francesca A, Anna IS (2014) Multiple effects of intracellular pH modulation in cancer cells. Cancer Cell Microenviron 1:e136. 10.14800/ccm.136

[58] Demetriou E, Story HE, Bofinger R, Hailes HC, Tabor AB, Golay X (2019) Effect of liposomal encapsulation on the chemical exchange properties of diamagnetic CEST agents. J Phys Chem B 123:7545–7557. 10.1021/acs.jpcb.9b02280 31449408 10.1021/acs.jpcb.9b02280 PMC6734798

