# Supplementary material for: Comparison of postprocessing metrics in multimetabolic APT-weighted CEST and 2-deoxy-D-glucose-CEST-MRI for differentiating breast cancer subtypes in a murine model
Source: Eur Radiol Exp. 2026 Jan 19;10:5. doi: 10.1186/s41747-025-00665-z (PMC12816453; doi:10.1186/s41747-025-00665-z)
Supplement: Supplementary file 1 — ELECTRONIC SUPPLEMENTARY MATERIAL [file 41747_2025_665_MOESM1_ESM.pdf]

Comparison of postprocessing metrics in multimetabolic APT-weighted CEST and 2-deoxy-D-glucose-CEST MRI for differentiating breast cancer subtypes in a murine model

ELECTRONIC SUPPLEMENTARY MATERIAL

Supplemental material

Methods: animal housing, welfare, and monitoring procedures

Female athymic BALB/c-derived nude mice (6–8 weeks) were purchased from Charles River Laboratories and acclimatized for 2 weeks before starting experiments. Mice were housed under controlled environmental conditions (21 °C ± 2 °C, 40–70 % humidity, 12-hour light/dark cycle) in groups of 7–8 per cage in individually ventilated cages (904 cm^2, Sealsafe Plus GR900, Techniplast, Germany). Mice were provided with bedding, nesting materials, chewing sticks, two red polycarbonate houses, and standard laboratory rodent diet and water *ad libitum*. Animal health and welfare were monitored daily using standardized health scoring sheets in accordance with institutional and national guidelines. Humane endpoints were predefined and strictly applied. Animals were euthanized if tumors exceeded a volume of 1500 mm^3, or if moist ulceration occurred. No animals exceeded these thresholds during the experiment.

Methods: statistics

Sample sizes were determined based on expected ΔMTR_{asym} enhancement in 2D-glucoCEST [53]. Based on an expected mean inter-subtype difference of 1.5 % and a within-group standard deviation of 1 %, pairwise post-hoc comparisons (Tukey's HSD) between three groups with a two-sided significance level of 0.05 require $n = 13$ animals per group to achieve 90 % power (effect size = 1.5). Allowing for a possible dropout rate of 20 %, the target sample size was set to $n = 16$–17 per group. Other CEST metrics (MTR_{REX}, AREX and APTw-CEST) were analyzed exploratively, and no formal sample size estimation could be performed.

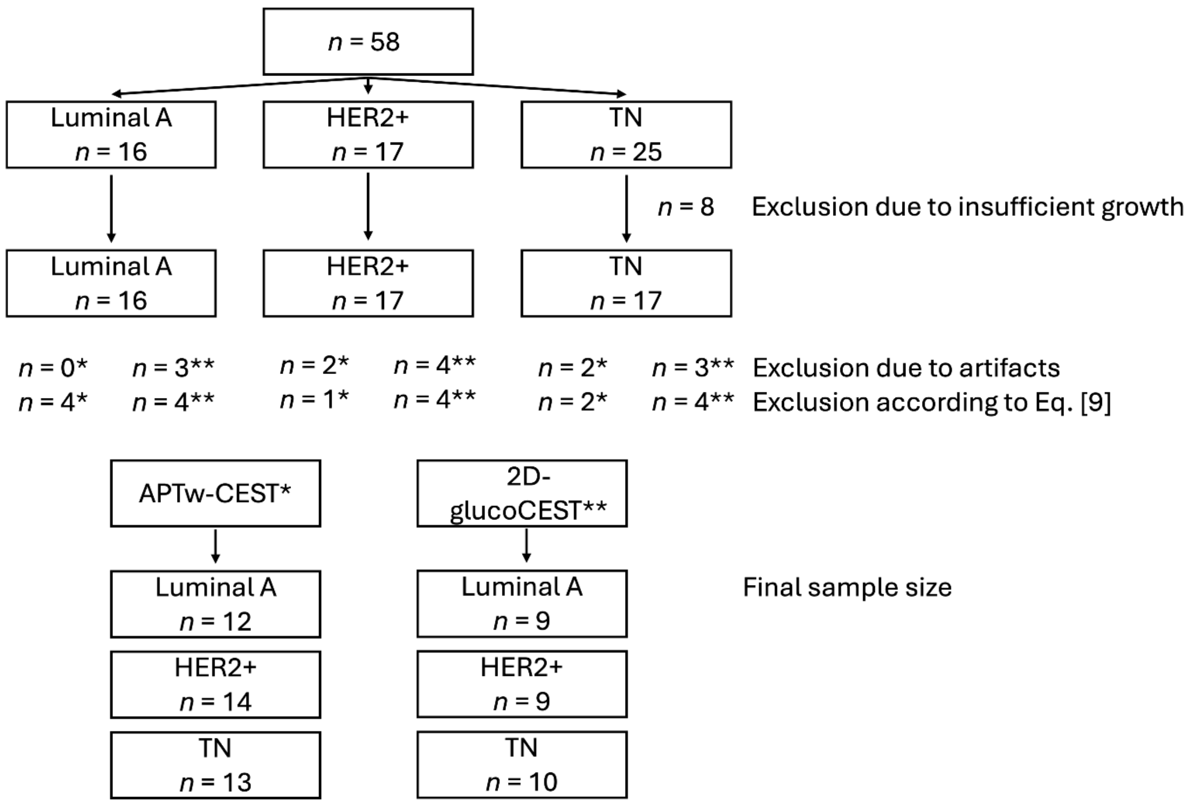

Fig. S1. Flowchart of initial animal numbers, exclusions, and criteria. Power calculations determined *n* = 15 per group; 1–2 extra mice were included to account for potential losses, which did not occur. Additional triple-negative mice were added after insufficient tumor growth in the first inoculation, yielding 17 successfully engrafted animals. Motion artifacts affected the two imaging methods differently, being more pronounced in 2D-glucoCEST due to the proximity of the exchange pool to water. No animal was excluded due to poor health.
Eq. [9]: corresponds to equation [9] in the Methods Section; * Excluded from APTw-CEST; ** Excluded from 2D-glucoCEST. *2D-glucoCEST* 2-deoxy-D-glucose Chemical exchange saturation transfer, *APTw-CEST* Amide proton transfer weighted-Chemical exchange saturation transfer, *HER2*+ Human epidermal growth factor receptor 2+.

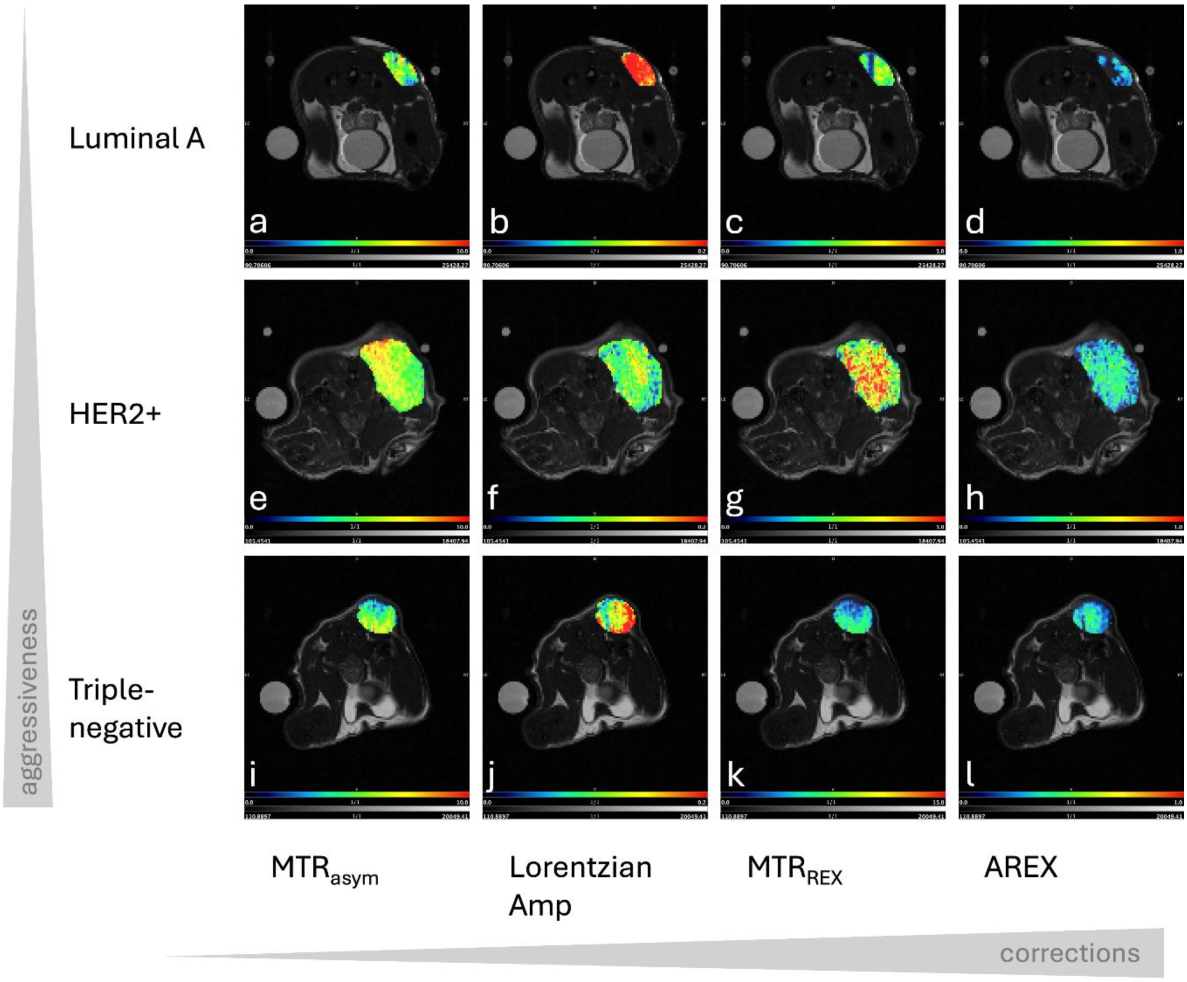

Fig. S2. T2-weighted anatomical images in axial orientation with a phantom on left and tumor at the dorsal side, overlapped with 2D-glucoCEST parametric maps. The same tumors as in **Fig. 4** are shown here. The first row shows a Luminal A tumor the second row a HER2+ tumor and the third row a triple-negative tumor. The following parametric maps are shown: **(a, e, i)** MTR_{asym}, **(b, f, j)** Lorentz amplitude, **(c, g, k)** MTR_{REX} and **(d, h, l)** AREX. *AREX* Apparent exchange-dependent relaxation, *MTR_{asym}* Magnetization transfer ratio asymmetry, *MTR_{REX}* Magnetization transfer ratio relaxation exchange
