# Supplementary material for: Comparison of postprocessing metrics in multimetabolic APT-weighted CEST and 2-deoxy-D-glucose-CEST-MRI for differentiating breast cancer subtypes in a murine model
Source: Eur Radiol Exp. 2026 Jan 19;10:5. doi: 10.1186/s41747-025-00665-z (PMC12816453; doi:10.1186/s41747-025-00665-z)
Supplement: Supplementary file 2 — Supplementary Tables [file 41747_2025_665_MOESM2_ESM.docx]

**Table S1.** Boundaries and starting conditions for Lorentzian fitting. Parameters for the 5-pool model are provided for APTw-CEST. Values were both chosen based on literature and visual inspection of the goodness of fit *in vivo*. All values are given in [ppm] for offset, [a.u.] for amplitude and [ppm] for width.

| Pool | Parameter | Lower bound | Center | Upper bound |
| --- | --- | --- | --- | --- |
| Water | Offset | -1 | 0 | 1 |
|  | Amplitude | 0.02 | 0.9 | 1 |
|  | Width | 0.3 | 1.4 | 10 |
| Amide | Offset | 3 | 3.5 | 4 |
|  | Amplitude | 0 | 0.025 | 0.2 |
|  | Width | 0.4 | 0.5 | 4 |
| NOE | Offset | -4.5 | -3.5 | -2 |
|  | Amplitude | 0 | 0.02 | 0.4 |
|  | Width | 1 | 7 | 5 |
| MT | Offset | -4 | -2 | 4 |
|  | Amplitude | 0 | 0.1 | 1 |
|  | Width | 10 | 25 | 100 |
| Amine | Offset | 1 | 2.2 | 2.5 |
|  | Amplitude | 0 | 0.01 | 0.2 |
|  | Width | 0.4 | 1 | 2.5 |

**Table S2.** Boundaries and starting conditions for Lorentzian fitting. Parameters for the 5-pool model are provided for 2D-glucoCEST. Values were both chosen based on literature and visual inspection of the goodness of fit *in vivo*. All values are given in [ppm] for offset, [a.u.] for amplitude and [ppm] for width.

| Pool | Parameter | Lower bound | Center | Upper bound |
| --- | --- | --- | --- | --- |
| Water | Offset | -1 | 0 | 1 |
|  | Amplitude | 0.02 | 0.9 | 1 |
|  | Width | 0.3 | 1.4 | 10 |
| B (0.66 ppm) | Offset | 0.4 | 0.3 | 0.7 |
|  | Amplitude | 0 | 0.05 | 0.1 |
|  | Width | 0.1 | 0.1 | 0.4 |
| D (1.28 ppm) | Offset | 0.9 | 1 | 1.5 |
|  | Amplitude | 0 | 0.1 | 0.2 |
|  | Width | 0.5 | 0.5 | 1 |
| E (2.08 ppm) | Offset | 1.5 | 1 | 2.5 |
|  | Amplitude | 0 | 0.2 | 0.3 |
|  | Width | 1 | 0.5 | 2 |
| F (2.88 ppm) | Offset | 2.2 | 2.9 | 3 |
|  | Amplitude | 0 | 0.2 | 0.2 |
|  | Width | 1 | 1 | 1.5 |

**Table S3.** Tumor volumes and cross-sectional tumor areas (corresponding to the same slice used for CEST analysis) were derived from ROIs drawn on T2-weighted anatomical images (mean $\pm$ standard deviation).

| **Breast cancer subtype** | **Tumor volume [mm^3^]** | **Slice [mm^2^]** |
| --- | --- | --- |
| Luminal A | 94.4 $\pm$ 61.6 | 2.2 $\pm$ 1.4 |
| HER2+ | 380.5 $\pm$ 307.8 | 5.1 $\pm$ 2.0 |
| Triple-negative | 303.9 $\pm$ 190.2 | 4.2 $\pm$ 2.2 |
| Total | 259.6 $\pm$ 240.2 | 3.8 $\pm$ 2.2 |

Note that MRI-derived volumes are lower than palpation-based estimates (diameter of ~1 cm corresponds to ~520 mm^3^) as skin and adipose tissue are excluded. Typical longitudinal growth rates can be found in literature for MCF-7 [1], SKBR-3 [2] and MDA-MB-231 [3] xenografts and show good agreement with our data.

**Table S4.** IQR for each quantification metric across BC subtype groups for both APTw-CEST and $\Delta$2D-glucoCEST.

| APTw-CEST | | | | |
| --- | --- | --- | --- | --- |
|  | MTR_asym_ [%] | Lorentz amplitude [a.u.] | MTR_REX_ [a.u.] | AREX [a.u.] |
| Luminal A | 2.65 | 0.0395 | 0.0163 | 0.00765 |
| HER2+ | 1.78 | 0.0267 | 0.0803 | 0.0424 |
| Triple-negative | 0.688 | 0.0355 | 0.0964 | 0.0401 |
| $\Delta$2D-glucoCEST | | | | |
|  | $\Delta$MTR_asym_ [%] | $\Delta$Lorentz amplitude [a.u.] | $\Delta$MTR_REX_ [a.u.] | $\Delta$AREX [a.u.] |
| Luminal A | 0.411 | 0.586 | 3.08 | 1.32 |
| HER2+ | 1.03 | 0.429 | 3.60 | 0.702 |
| Triple-negative | 0.862 | 0.767 | 1.69 | 0.962 |

*2D* 2-deoxy-D-glucose, *APTw* Amide proton transfer weighted, *AREX* Apparent exchange-dependent relaxation, *CEST* Chemical exchange saturation transfer, *MTR_asym_* Magnetization transfer ratio asymmetry, *MTR_REX_* Magnetization transfer ratio relaxation exchange.

1. Dall G, Vieusseux J, Unsworth A, Anderson R, Britt K (2015) Low dose, low cost estradiol pellets can support MCF-7 tumour growth in nude mice without bladder symptoms. J Cancer 6:1331–1336. https://doi.org/10.7150/jca.10890

2. Sokolova EA, Proshkina GM, Kutova OM, Balalaeva IV, Deyev SM (2017) The effect of the targeted recombinant toxin darpin-pe40 on the dynamics of her2-positive tumor growth. Acta Naturae 9:103–107

3. Lim HK, Lee H, Moon A, Kang K-T, Jung J (2018) Exploring protocol for breast cancer xenograft model using endothelial colony-forming cells. Transl Cancer Res 7:1217–1224. https://doi.org/10.21037/tcr.2018.09.09
